# 

**Published:** 1861-01

**Authors:** 


### INDEX TO VOLUME II.

PAGE.
A Medical Sharp-Shooter,............ 377
A New Mode of Counter-Extension. By E. ANDREWS, M. D., etc........... . 659
Absence of the Uterus,................ 377
Acromion Process, Fracture of......... 300
Actæa Racemosa, ......... ......... 356
Action of Chloroform on the Blood,.... 431
Action of Opium on the Genito-Urinary Organs. By B. WOODWARD, M. D., Galesburg, Ill.,................. 473
——By GEO. B. WILLSON, M. D., Pt. Huron, Mich., ....... ............. 563
Acute Articular Rheumatism.......... 170
Acute Pleurisy,...................... 341
Ague Cake. A Clinical Lecture, by W. H. BYFORD, M. D., Chicago, Ill.,.... 415
Alcoholic Drinks, their influence on the development and progress of Tuberculosis. By N. S. DAVIS, M. D.,.... 65
——Cleveland Medical Gazette, *vs*..... 368
Alcoholic Stimulants in Hospitals...... 432
Amaurosis,.......................... 340
Amputation of the Cervix Uteri. By J. MARION SIMS, M. D., New York,..... 621
American Medical Association,........58–127
Anæsthesia, Rules for the Induction of. By Prof. S. D. GROSS, M. D., Phila. 288
——The Miracle of. By FRANK W. REILLY, M. D., Chicago, Ill.,....... 269
Anæsthetics. A Letter from DAVID PRINCE, M. D., Jacksonville, Ill.,.... 122
——in Midwifery. By Dr. B. FORDYCE BARKER .............................. 674
——Kerosolene,.................. .. 406
——Theory of their Action. By C. T. JACKSON, M. D., .................. 428
——Turpentine, ........ ............, 245
ANDREWS' New Splint for Hip Disease, 294, 444
Aneurism of the Aorta,................ 297
Army Medical Society at Cairo,......... 692
Army Sanitary Commission,......374, 565, 689
Army Surgeons, Hints to... .......... 542
Aromatic Sulphuric Acid for Tape-Worm 126
Arsenious Acid, a Substitute for Quinine, 571
Arsenical Poisoning, Cases of. By N. S. DAVIS, M. D.,........................ 421
Arseniate of Gold in Cancer,.......... 126
——Of Soda in Scrofula,.............. 425
Art and Nature in the Management of Disease. By N. S DAVIS, M. D., etc., 129
Articular Rheumatism,....... ....... 170
Auscultation, Drill for ............... 482

Belladonna Shortening Labor, ......... 701
Bellevue Hospital College,.... ........ 237
Burns, Stimulating Treatment of....... 671

PAGE.
*Book Notices:*
BARWELL: On the Joints,............. 551
BEDFORD: Principles and Practice of Obstetrics, ........................ 685
BILLROTH: On Tumors,....... ....... 363
BROWN-SEQUARD: On Paralysis,....... 495
BUMSTEAD: On Venereal,............558, 634
DALTON: Human Physiology,......... 227
GARRATT: Electro-Physiology and Therapeutics,.......................... 181
GIBBS, O. C.: On Cholera,............ 315
GIBBS, R. W. Jr.: Registration Report of So. Ca.,......................... 46
GROSS, S. D., American Medical Biography,..... ..................... 182
——Manual of Military Surgery,....... 360
HAMILTON: Military Surgery,.......... 360
HILLES: Pocket Anatomist,.. ........ 47
HODGES: Diseases Peculiar to Females,. 46
LAWSON: On Phthisis Pulmonalis,....181, 307
LEIDY: Human Anatomy,............46, 180
LYONS: On Fevers,.... ............. 229
MAXSON: Practice of Medicine,......... 434
MORLAND: On Uræmia,.... .......... 557
O'REILLY: On Baths, Heat, Cold, etc., 366
READ: Placenta Prævia............... 681
SANFORD: The Gorilla ................ 683
SAYRE: On Morbus Coxarius, ........ 229
SLADE: On Diphtheria, ...... ...... 183
SWINBURNE: On Extension in Treatment of Fractures,.... .. ............... 554
TAYLOR: Movement Cure,............ 183
THOMPSON: On Fever,.... ............ 184
TRIPLER & BLACKMAN: Hand-book for the Military Surgeon,............. .. 314
WATSON: The True Physician,........ 367
WELLS: Summary of Medical Science,. 230
WEST: Diseases of Women............ 677
WOODWARD: Address before Conn. Med. Society,.... ...................... 500
WYNNE: On Diphtheria,............... 231
WYTHE: Pocket Dose and Symptom Book,.... ......................... 559
——Exchanges,....................... 435
——Berkshire Medical Journal,........ 187
——BRAITHWAITES' Retrospect,.... ... 435
——Buffalo Med. & Surg. Jour. & Rep., 500
——HAY'S Journal Med. Science,...... 347
——London *Lancet*, .., ....... 367, 436, 500
——N. A. Medico-Chirurgical Review,. 346
Report of Board of Health of Philadelphia for 1860,.......................... 313
——Of Chicago Charitable Eye and Ear Infirmary,....................... 362

PAGE.
Report of a Com. of the Boston Med. Soc. on Sulphuric Ether, 625
——Of Friends' Asylum, 312
——Of Longview, O., Insane Asylum.. 313
Transactions of Indiana State Med. So., 501
——New Jersey State Med. Soc., 368
——Ohio State Med. Soc., 226

Cæsarian Section, 49
Calculus, a Clinical Lecture on. By Prof. E. ANDREWS, 476
Cancer, Arseniate of Gold in 126
Cannabis Indica, 381
Caoutchouc in Phthisis, 359
Case of Extra-Uterine Pregnancy, etc., By E. P. COOK, M. D., Mendota, Ill., 458
Case of Periodical Strabismus. By F. N. SMITH, M. D., Laharpe, Indiana, 532
Cases of Arsenical Poisoning. By N. S. DAVIS, M. D., Chicago, Ill., 421
Catalepsy, Chloroform in. By Dr. A. WOODWARD 666
Cataract, Double Congenital, 510
Causes of Mild Stomatitis in Infants, 572
Cervix Uteri, Amputation of 621
Chancre, HAMMOND on 548
Changes in Medical Colleges in this City 503
Chicago Academy of Medical Sciences, 63
——*Medical Journal* and Lind University, 501
——*Med. Examiner* for 1862 686
——Sanitary Commission, 562
Chlorate of Potassa in Phthisis, 245
——in Variola, 379
——in Stomatitis Materna, 564
——and Glycerine as a Dressing, 574
——Some of its Uses 667
Chloroform in Congestive Chills, 127
——in Catalepsy. By ASHBEL WOODWARD, M. D., Franklin, Conn., 666
——Its Action on the Blood, 431
——To Prevent Vomiting from 291
——and Iodine in Ozœna, 610, 613
Cholera Infantum and Teething, 438
Chorea, Treatment of 52
——Cimicifuga Rac. in 331, 356
Cimicifuga Racemosa. SIMPSON, PARRISH and DAVIS, 331
——Dr. J. Y. SIMPSON, 356
Cleveland *Med. Gazette*, *vs.* Dr DAVIS' Paper on the Influence of Alcohol in Phthisis, 368
Clinical Instruction and Med. Schools 437
Clinique, The (See *The Clinique*.)
Close of the Volume, 639
Co-existence of Diseases, 378
Coffee as a Remedy for Whooping Cough 433
Comments on Prof. BRAINARD'S "Extirpation of the Parotid." By TITUS DEVILLE, M. D., Stockport, Eng., 264
Congestive Chills, Chloroform in 127
Conservative Surgery. By H. WANZER, M. D., Chicago, Ill., 338
——NAPIER'S Case, 378
Consumption of Alcoholic Stimulants in Hospitals, 432
Contagiousness of Diphtheria, 317
Counter-Extension, a New Mode of. By Prof. E. ANDREWS, M. D., etc 659
Coxalgia Threatening on Recovery from Typhoid Fever, 350
Cure for Whooping-Cough 696

Death from Prolonged Irritation of the Left Phrenic Nerve. By Prof. J. H. HOLLISTER, M. D., Chicago, Ill... 327
Delirium Tremens, 51, 127, 574

PAGE.
Demonstrator of Anatomy 562
DEVILLE on Prof. BRAINARD'S "Extirpation of the Parotid," and Treatment of Spina Bifida, 264
Diarrhœa and Dysentery, Opium and Nitric Acid in 673
Digitalis in Delirium Tremens, 127, 574
——In Heart Disease, 48
Dilute Glycerine, 583
Diphtheria, A Clinical Lecture on. By Prof. N. S. DAVIS, Chicago, Ill., 153
——An Essay on. By T. S. PEARCE, M. D. Decatur, Ind., 36
——By HENRY W. DAVIS, M. D., Paris, Illinois, 79
——Its Nature and Treatment. By C. W. DAVIS, M. D., Indianola, Iowa., 150
——Contagiousness of, 317
——Per-chloride of Iron in, 116
Directions to Surgeons on the Battle Field 302
Diseases, co-existence of 378
Dislocation of Humerus of 14 weeks standing 480
Drill for Auscultation 482
Dropsical Fluid, Large amount of, 696
DUPUYTREN on Spurious Pregnancy, 696

*Editorial :*

A Report on Medical Education 118
A Suggestion 242
An Explanation 231
Action of Opium on the Genito-Urinary Organs 563
American Medical Association 58
——Postponement of Meeting 237
Army Medical Society at Cairo, 692
Army Sanitary Commission 374, 565, 689
Berkshire Medical Journal 187
Bellevue Hospital College 237
Change of Name 187
Changes in Medical Colleges in Chicago 503
Chicago *Medical Examiner* for 1862, 686
Chicago *Med. Journal* and Lind Univ.. 501
Chicago Sanitary Commission 562
Cholera-Infantum—Summer Complaints and Teething 438
Clinical Instruction and Medical Schools 437
Cleveland *Medical Gazette* vs. Dr. DAVIS' Paper on the Influence of Alcohol in Phthisis 368
Contagiousness of Diphtheria 317
Demonstrator of Anatomy 562
Further Experiments with Kerosolene.. 505
Homœopathy in Michigan University 188
——Letter from Dr. PALMER on 238
——Renunciation of 504
Illinois State Medical Society 185
Kerosolene, Further Experiments 505
Mal-Practice Suit 561
Medical College Commencements in Chicago 185
——of Lind University 186
——Summer Course of 127, 188
——and *Chi. Med. Journal*, 501
Medical Education, A Report on 118
Medical Societies 559
Mortality of Chicago, 695
New Sydenham Society 189
New York Ophthalmic School 187
Opening of Medical Colleges in Chicago 563
Prospects for the Future 231
Report of Board of Medical Examiners to the State Legislature 233
——on Medical Education 118

PAGE.
Resignation of Prof. MEIGS............ 240
Scurvy.... ... ......................... 319
Summer Complaint and Teething...... 438
Summer Course of the Lind Medical Department.........................127, 188
Summer Schools......... . ........... 317
Surgeons and Asst. Surgeons in the Army 232
——for the Illinois Volunteers.......... 446
——to Marine Hospital................. 236
Transactions of the Ills. State Medical Society.......:................... ... 60
Volunteer Women Nurses.............. 376
Western Army Medical Matters......... 372
What ails them?..... ................. 560
Work on New Remedies,.............. 693
Effects of Climate on No. & Sou. Troops, 572
Enteric Fever......................... 491
Epilepsy, Hyprocyanate of Iron in, ... 53
Epulis, Malignant...................... 413
Ergot, its History, &c................. 93
Erythema .................. ........ 343
Examining Board for Army Surgeons... 232
Excision of Nerves in Facial Neuralgia, A Clinical Lecture, by E. ANDREWS, M. D.,........... ................... 148
Excision of Head of Femur in Hip-Joint Disease, Clinical Remarks by E. ANDREWS, M. D.,..................... 292
Explanation, An ....... ............. 231
Extirpation of the Eye. By E. L. HOLMES, M. D.,.................... 655
Extra-Uterine Pregnancy continuing 4 years. By E. P. COOK, M. D., Mendota, Ill.......... ............ 458
Extracts from Correspondence:—Dr. S. W. WALLACE,....................... 690
——:—"G. G. S.," New York,........ 691
——: Dr. C. DUMREICHER, Wheeling, Va 691
Facial Neuralgia. A Clinical Lecture, by E. ANDREWS, M. D.,............... 71
Fever, Enteric......................... 491
Fistula from the Hip-Joint into the Rectum .... ......................... 293
Fracture of Acromion Process, Clinical Remarks by RALPH N. ISHAM, M. D., in Marine Hospital......... ........ 300
Fracture of Spine. By GEO. K. AMERMAN, M. D., Chicago, Ill............. 406
Fracture Splints of Tin................ 475
Fractures of the Leg,.................. 670
Fragilitas Ossium. By ORRIN SMITH, M. D., Chicago, Ill.,.................. 584
French Surgery in the Crimea.......... 494
Further Experiments with Kerosolene. By FRANK W. REILLY, M. D. Chicago, 505
Gastritis. A Clinical Lecture, by N. S. DAVIS, M. D.......... ............ 202
Gastrotomy in case of Extra-Uterine Pregnancy ......................... 458
Genito-Urinary Organs, Action of Opium on ...............................473, 563
Glycerine, dilute... .................. 583
Glycerole of Chlorate of Potassa...... 574
Gonorrhœa, Military Hospital treatment of .................. ....... .. 506
Gunshot Wounds ... .................. 607
Gunshot Wounds produced during the Loading of Artillery,................ 697
GUTHRIE'S Directions to Surgeons on the Battle Field........................ 302
HAMMOND on Chancre................. 548
Heroism of Army Surgeons ..........508, 573
Hernia, Triple......................... 419
——Operation for................... ... 481
——Strangulated.................523, 193, 671
Hints to Army Surgeons................ 542
Hip-Joint Disease—A New Splint for... 294
—— Clinical Remarks on, by E. ANDREWS, M. D......................... 292
—— Fistula into Rectum from.......... 350
—— Mercurial Treatment of. By W. GODFREY DYAS, F. R. C. S., Chicago, 598
Homœpathy in Mich. University... ... 188
—— Letter from Prof. A. B. PALMER, M. D., Detroit, Mich..................... 238
—— Renunciation of, by Dr. J. C. PETERS ...............................488, 504
Housemaids' Bursæ,.................. 673
Humerus, Dislocation of................ 480
Hydrocyanate of Iron in Epilepsy...... 53
Hygiene, Public and Legal Medicine. .. 1
Hysteria,............................. 673
Industry of JOHN HUNTER............. 571
Inhalations in the Treatment of Inflammations of the Nasal Passages, and their Appendages. By FRANK W. REILLY, M. D..... ...................... 613
Injection of Iodine in Spina Bifida. BRAINARD, NELATON and DEVILLE... 264
Intermittent Fever, Nitric Acid in ..... 243
Inversio-Uteri, its Causes, Mechanism, and Medico-Legal Bearings. By N. S. DAVIS, M. D. ........ .......... . ... 4
—— A Case of. By R. C. HAMILL, M.D. Chicago ............................. 207
—— Discussion on, in N. Y. State Med. Society............................ 212
Iodide of Ammonium, a Substitute for Potassium Iodidii........ ............ 117
—— of Iron in Phthisis................ 178
Iodine and Chloroform in Ozœna. A Clinical Lecture by Prof. WM. H. BYFORD, M. D......................... 610
Kerosolene, an Experiment with. By E. ANDREWS, M. D.................... 406
—— Experiments with. By Dr. J. H. BIGELOW........................... 408
—— Further Experiments. By FRANK W. REILLY, M. D ................... 505
—— Letter upon, from WM. DICKINSON, M. D................................ 439
Lectures on Military Surgery. By E. ANDREWS, M. D. Professor of Surgery, etc....249, 257, 321, 385, 449, 513, 577
Lind University, Med. Dept., Summer Course of,......... ..........127, 188, 317
—— Second Annual Commencement.... 186
—— Third Winter Session........ ... 638
Lithotomy. A Clinical Lecture and Operation. By E. ANDREWS, M. D., etc 533
Lithotrity,. .... ............... .... 671
Local Medical Societies... ............ 639
Mal-Practice Suit...................... 561
Malt, Powdered, as Food for Children.. 245
Mania-a-Potu. Treatment of........... 51
Maxims of Military Surgery ........... 246
Measles, A Clinical Lecture on......... 344
—— Predisposing to Fevers............ 572
Medical Department, Lind University, 127, 186, 188, 317, 638
Medical Education, a Report on........ 118
Medical Schools and Clinical Instruction 437
Medical Sharp-Shooter................ 377
Medical Societies..................... 559
Medical Societies, Local........ ....., 639
Memoirs of MARSHALL Hall............ 616
Mercurial Treatment of Morbus Coxæ. By GODFREY DYAS, F.R.C.S......... 598
Method of Shortening Tedious Labors.. 49
Mild Stomatitis in Infants.............. 572

PAGE.
Military Hospital Treatment of Gonorrhœa.... 506
Military Surgery, GUTHRIE'S Rules..... 302
—— Lectures on. By E. ANDREWS, M. D., Prof. of Surgery, etc........ 249, 257, 321, 385, 449, 513, 577
—— STROMEYER'S .... 246
Milk Sickness. GEO. FISHER, Morris, Ill. 539
—— JOHN A. KENNICOTT, M. D. The Grove, Ill.... 538
Miracle of Anæsthesia. By FRANK W. REILLY, M. D., Chicago, Ill.... 269
Mortality among Medical Journals,.... 695
Mortality of Chicago,.... 695
Muriate of Ammonia, Therapeutical Uses of,.... 649
Narrow and Irritable Strictures of the Urethra .... 221
Nature and Art in the Management of Disease. By N. S. DAVIS, M. D., Prof. Practical Medicine, etc........ 129
Nerve-Fibre, Regeneration of.... 380
Neuralgia, Facial. By E. ANDREWS, M. D., etc.... 71
—— Excision of Infra-Orbital Nerve in.. 148
—— Sciatic, Clinical Remarks on...... 166
New Applications of the Per-Chloride of Iron,.... 694
New Splint for Hip-Joint Disease....... 294
New Sydenham Society .... 189
Nipple—Benzoin for Chapped.... 50
Nitric Acid and Opium in Diarrhœa and Dysentery,.... 673
Nitric Acid as an Anti-Periodic.... 243
Nurses, Volunteer Women.... 376

Obituary: Sir JOHN FORBES.... 693
Dr. E. J. FOUNTAIN .... 236
Dr. JOHN W. FRANCIS...... 191
HENRY GRAY.... 376
Dr. THOS. HARRIS .... 241
Surgeon-Gen. LAWSON...... 318
Dr. D. M. REESE.... 318
M. SCRIVE.... 693
Oil of Valerian in Typhus.... 573
On the Therapeutical Employment of the Oxide of Zinc. By Dr. S. WATERMAN. 545
"Onward!".... 377
Opening of Medical Colleges in Chicago. 563
Opium, its Action on the Genito-Urinary Organs. By B. WOODWARD, M. D., Galesburg, Ill.... 473
—— By GEO. B. WILLSON, M. D., Pt. Huron, Mich.... 563
Opium and Nitric Acid in Diarrhœa and Dysentery,.... 673
Out-Door Life for Phthisis.... 51
Ovarian Tumors. By WM. H. BYFORD, M. D., Prof, of Obstetrics, etc.,....... 641
Ovariotomy, Justifiableness of. By WM. H. BYFORD, M. D., etc.,.... 641
Oxalate of Cerium in Vomiting.... 50
Oxide of Zinc.... 545
Ozœna, A Clinical Lecture. By WM. H. BYFORD, M.D., Prof. of Obstetrics, etc 610
—— Iodine and Chloroform in. By FRANK W. REILLY, M. D.... 613

Papular Eruption. Dr. ISHAM'S Clinic. 297
Pelvic Measurements. By WM. H. BYFORD, M. D.... 112
Perchloride of Iron in Diphtheria...... 116
——New Applications of,.... 694
Periodical Strabismus, a Case of. By F. N. SMITH, M. D., Laharpe, Ill........ 532
Periosteal Reproduction of Bone,...... 698

PAGE.
Phrenic Nerve, Death from Prolonged Irritation of. By J. H. HOLLISTER, M. D. .... 327
Phthisis, Caoutchouc in.... 359
—— Chlorate of Potash in.... 248
—— Iodide of Iron in.... 178
—— Out-Door Life for .... 51
Physical Training,.... 696
PIROGOFF'S Operation.... 567
Pityriasis Versicolor. Dr. ISHAM'S Clinic 297
Placenta Prævia. By J. H. APPERSON, M. D., Bourbon, Ill. .... 339
Placenta, Removal of. CREDE.... 357
Pleurisy, Acute.... 341
Podophyllin as a Substitute for Mercury 697
Position a Remedy for Stertor.... 117
Powdered Malt for Children's Food..... 245
Pregnancy, Extra-Uterine. By E. P. COOK, M. D., Mendota, Ill.... 458
Proceedings of Societies: Chicago Academy of Medical Science.... 162
—— Chicago Medical Society.........424, 239
—— De Witt Co. Med. Society........420, 165
—— Esculapian Society at Paris, Ill.... 54
—— Will Co. Med. Society.... 164
Prolapse of the Funis and Craniotomy. By F. N. SMITH, M. D., Laharpe, Ill.. 76
Prolapsed Bowel Treated by Subcutaneous Strychnia Injections.... 434
Prolapsus Uteri with Ulceration. By D. D. WAITE, M. D., Chicago, Ill.... 397
Prophylactic use of Quinine.... 567
Prospects for the Future.... 231
Public Hygiene and Legal Medicine, a Report on. By H. W. JONES, M. D., Chicago. Ill.,.... 1
Puerperal Convulsions,.... 702

Quinine as a Prophylactic.... 567

Recording Medical Practitioners....... 243
Regeneration of Nerve-Fibre.... 380
——of Osseous Tissue,.... 700
Removal of Placenta.... 357
Renunciation of Homœopathy.... 488
Reorganization of the Medical Department of the Army,.... 699
Report of Board of Medical Examiners to State Legislature .... 233
—— of Committee on Public Hygiene & Legal Medicine.... 1
—— On the Sanitary Condition of the City of Chicago. By N. S. DAVIS, M. D.........528, 661
Rheumatism, Acute Articular.... 170
—— Cimicifuga Racemosa in.........331, 356
—— By B. WOODWARD, M. D., Galesburg, Ill.... 353
Rubeola, A Clinical Lecture. By N. S. DAVIS, M. D .... 344
Rules for the Induction of Anæsthesia. By S D. GROSS, M. D., Prof. of Surgery, etc.... 288
Rupture of the Uterus. By JAS. S. KING. M. D., Lemont, Ill.... 520
Rush Medical College.... 639
Russell on American Military Surgeons. 573

Sanitary Condition of Chicago, a Report on, By N. S. DAVIS, M. D. ......528, 661
Sanitary Commission..........562, 565, 689
Sciatic Neuralgia.... 166
Scrofula, Arseniate of Soda in.... 425
Scurvy.... 319, 330
—— In the Army of the Mississippi..... 637
Smithsonian Institution.... 117

PAGE.
Spina Bifida. By VINDEX............. 403
——Injections of Iodine in. BRAINARD, NELATON and DEVILLE............... 264
Spinal Curvature. A Clinical Lecture. By N. S. DAVIS, M. D............... 38
Spine, Fractures of. By G. K. AMERMAN Chicago, Ill ........................ 409
Splint, ANDREW's, for Hip-Joint Disease ...............................294, 444
Splints, Tin Fracture.................. 475
Steadine.............................. 244
Stertor, Postural Treatment of......... 117
Stomatitis Materna, Chlorate of Potassa in.................................. 564
Strabismus, Periodical. By F. N. SMITH Laharpe, Ill......................... 532
Strangulated Hernia. By ALEX. FISHER M. D., Chicago, Ill. ................. 193
—— By W. A. GORDON, M. D., Wausau, Wis.................................. 523
——By F. C. SKEY, F.R.C.S.,......... 671
Stimulating Treatment of Burns,....... 671
Strictures, Irritable, of the Urethra. SLADE............................. 221
STROMEYER'S Maxims.................. 246
Strychnine, Tannin an Antidote to.... 301
—— Subcutaneous Injections of........ 434
Successful Operation for Double Congenital Cataract ...................... 510
Suggestion, A......................... 242
Summer Complaint and Teething....... 438
Summer Medical Schools,.........127, 188, 317
Superfœtation. By J. H. APPERSON, M. D., Bourbon, Ill.,.................... 339
Surgeons for Volunteer Forces,.........232, 442
Surgeon to Marine Hospital,.......... 236
Surgery, Conservative. By H. WANZER, M. D. Chicago, Ill.,................. 338
——NAPIER'S Case,................... 378
Surgical Notes from the *Lancet*........ 670
Syphilitic Rupia. Dr. ISHAM'S Clinic,.. 342

Tannin an Antidote for Strychnia,..... 301
Tape-Worm, Aromatic Sulphuric Acid, for,.................................. 136
Tartar Emetic in Asthma.............. 703
Tedious Labor, Method of shortening,.. 49
Teething and Cholera Infantum,........ 438
*The Clinique :*
Acute Pleurisy. Service RALPH N. ISHAM M. D., Surgeon to Marine Hospital.. 341
Ague Cake. Service WM. H. BYFORD, M. D., Prof. of Obstetrics and Diseases of Women and Children.............. 415
Aneurism of Aorta with Cardiac Hypertrophy. Service Dr. ISHAM.......... 297
Calculus. Service E. ANDREWS, A. M., M. D., Prof. of Clinical Surgery...... 476
Chronic Gastritis; Post Mortem Appearance, etc. Service N. S. DAVIS, M.D. Prof. of Clinical Medicine........... 202
Chronic Papular Eruption. Service Dr. ISHAM ............................... 295
Complete Amaurosis. Service Dr. ISHAM 340
Coxalgia supervening on Recovery from Typhoid. Service Dr. DAVIS........ 350
Diphtheria. Service Dr. DAVIS........ 153
Dislocation of Humerus. Service Dr. ISHAM .............................. 480
Drill for Ausculation. Service THOS. K. CHAMBERS, M. D................... 482

PAGE.
Erythma. Service Dr. ISHAM........... 343
Excision of Head of Femur. Service Dr. ANDREWS......................... 292
Facial Neuralgia. Treatment by Subcutaneous Injections and by Excising Nerve. Service Dr. ANDREWS........ 71
—— Excision of Infra-orbital nerve. Service Dr. ANDREWS................ 148
Fistula from the Hip-Joint into the Rectum. Service Dr. ANDREWS ......... 293
Fracture of the Acromion. Service Dr. ISHAM............................... 300
Gun-Shot Wounds. Service Dr. ANDREWS ................................ 607
Lithotomy. Service Dr. ANDREWS..... 533
Ozœna. Service Dr. BYFORD........... 610
Operation for Hernia. Service Dr. ISHAM 481
Operation for Removal of Malignant Epulis. Service Dr. ANDREWS....... 413
Pityriasis Versicolor. Service Dr. ISHAM 297
Rubeola. Service Dr. DAVIS.......... 344
Syphilitic Rupia. Service Dr. ISHAM... 342
Traumatic Spinal Curvature; Paraplegia and Anæsthesia. Service Dr. DAVIS .................................. 38
Triple Hernia. Service Dr. ANDREWS.. 419
Typhoid Fever, with threatened Coxalgia. Service Dr. DAVIS,............ 350
The Chicago *Med. Journal* and Lind University,.......................... 501
The Mercurial Treatment of Morbus Coxæ. By W. GODFREY DYAS, F.R.C.S., Chicago, Ill................... 598
The Miracle of Anæsthesia, An Inaugural Thesis. By FRANK W. REILLY, M. D., Chicago, Ill.,................ 269
The New Splint for Hip Disease. A Letter from Dr. E. ANDREWS,....... 444
The Surgeon. An Introductory Lecture, By E. ANDREWS, M. D., Prof. of Surgery, etc.,............................ 587
The Surgeons of the New York 8th,.... 508
The Therapeutical Uses of the Muriate of Ammonia. By M. O. HEYDOCK, M. D., Chicago, Ill.,................ 649
Theory of the Action of Anæsthetics,... 428
Thoracentesis,......................... 47
Three Cases of Extirpation of the Eye. By E. L. HOLMES, M. D., Chicago, Ill 655
Tin Fracture Splints,.................. 475
Tincture Benzoin for Chapped Nipples, 50
Tobacco, Prof. MILLER on,............ 174
——Smoking,........................ 114
Tough Lunar Caustic Points,.......... 377
Transactions of Illinois State Med. Soc. 60
Transfusion of Blood in Obstetrical Practice,................................ 358
Treatment of Child-Bed Fever,........ 699
Triple Hernia,......................... 419
Tuberculosis; the Influence of Alcoholic Drinks in the Development and Progress of. By N. S. DAVIS, M. D. Chicago, Ill.,.......................... 64
Turpentine as an Anæsthetic,.......... 245
Typhoid Fever; etc., Clinical Remarks. By N. S. DAVIS, M. D.,.............. 350

United States Sanitary Com.....374, 565, 089
Urethra, Strictures of, SLADE,........ 221
Uterus, Absence of,.................. 377
——Amputation of Cervix of,.......... 621

PAGE.
Uterus, Inversion of, its Causes, etc. By N. S. DAVIS, M. D.,............... 4
——Prolapsus of, with Ulceration. By D. D. WAITE, M. D., Chicago, Ill.... 397
——Rupture of. By J. S. KING, M. D., Lemont, Ill.,........................ 520
Valedictory Address to the Graduating Class of 1860-61, Med. Dept. Lind University. By Prof. M. K. TAYLOR, M. D.,............................ 139
Valerian in Typhus,..................... 573
Valuable Donation,..................... 687
Variola, Chlorate of Potassa in, ...... 379
Veratrum Viride. By E. S. McINTIRE, M.D., Dallas City, Ind., ............. 200

PAGE.
Volunteer Women Nurses,........... ..... 376
Vomiting, Oxalate of Cerium in,........ 50
——from Chloroform, To Prevent,..... 291

WARDELL on Enteric Fever,........... 491
Western Army Medical Matters, ...... 372
What Ails Them?...................... 560
Whooping-Cough—Coffee as a Remedy, 433
——Clover Hay in,..................... 665
——Cure for........................... 696
Women Nurses, Volunteer,............ 376
Work on New Remedies,............... 693
Wounds into Joints,.................. 672

### GALE BROTHERS,
### PHARMACEUTISTS,

Dealers in

**SELECT DRUGS, RARE CHEMICALS, FRENCH ARTIFICIAL EYES, TRUSSES, SUPPORTERS, BRACES, ELASTIC STOCKINGS,**

**And almost every Article pertaining to the Trade,**

AGENTS FOR B. KEITH & CO.'S CONCENTRATED ORGANIC MEDICINES, AND C. PATUREL & CO.'S SWEDISH LEECHES.

**Prompt and Particular attention paid to Physician's Orders.**

202 Randolph Street, Chicago.

### THE CHICAGO
### MEDICAL EXAMINER.

N. S. DAVIS, M. D., . . . . . . . EDITOR.

PUBLISHED BY WM. CRAVENS & CO.

**132 LAKE-ST., CHICAGO.**

The EXAMINER will be issued during the first week of each month, commencing with January, 1860. Each number will contain 64 pages of reading matter, the greater part of which will be filled with such contents as will directly aid the practitioner in the daily practical duties of his profession.

To secure this object fully, we shall give, in each number, in addition to ordinary original articles, and seleetions on practical subjects, a faithful report of many of the more interesting cases presented at the Hospitals and College Cliniques. While aiming, however, to make the EXAMINER eminently practical, we shall not neglect either the scientific, social, or educational interests of the profession. It will not be the special organ of any one institution, society or clique. But its columns will be open for well written articles from any respectable member of the profession, on all topics legitimately within the domain of medical literature, science, and education.

**Terms, $2.00 per annum, invariably in advance.**

